# Tomato plants control leaf sodium levels to limit damage by *Spodoptera littoralis* larvae

**DOI:** 10.1111/nph.71150

**Published:** 2026-04-09

**Authors:** Valerio Cirillo, Ilaria Di Lelio, Paola Punzo, Giovanni Jesu, Maria Giovanna De Luca, Marco Cepparulo, Claudio Russo, Nausicaa Pollaro, Antonio Marciano, Andrea Becchimanzi, Michael V. Mickelbart, Francesco Pennacchio, Albino Maggio

**Affiliations:** ^1^ Department of Agricultural Sciences University of Naples Federico II Portici 80055 Italy; ^2^ Interuniversity Center for Studies on Bioinspired Agro‐Environmental Technology (BAT Center) University of Naples Federico II Portici 80055 Italy; ^3^ CNR Institute of Biosciences and Bioresources Research Division Portici Portici 80055 Italy; ^4^ Center for Plant Biology Purdue University West Lafayette IN 47906 USA; ^5^ Department of Botany and Plant Pathology Purdue University West Lafayette IN 47907 USA; ^6^ Department of Horticulture and Landscape Architecture Purdue University West Lafayette IN 47907 USA

**Keywords:** larvae, pest tolerance, plant–insect interaction, sodium, sodium translocation, *Solanum lycopersicum*, *Spodoptera littoralis*

## Abstract

- Sodium is an essential element for animal growth and survival. Here we test the hypothesis that plants exposed to insect feeding can translocate sodium from the feeding site to other plant organs as a defense strategy against phytophagous insects, which need sodium in their diet.
- By modulating leaf sodium levels in tomato and by profiling the gene expression of key plant sodium transporters in response to insect feeding, we assessed sodium redirection pathways as affected by leaf damage and the impact of different sodium levels on food preference and intake, growth and development of *Spodoptera littoralis* larvae.
- We demonstrated that upon *S. littoralis* attack, tomato plants removed sodium from the feeding site by relocating it basipetally from the leaf to the root, through a coordinated regulation of the sodium transporters *HKT1;1* and *HKT1;2*. We also showed that the leaf damage by *S. littoralis* larvae was positively correlated with sodium concentration and that low sodium plants negatively affected food preference, larvae growth and development.
- These results shed light on a novel mechanism of plant response to herbivore insect damage, providing new insights on the role of sodium in plant–insect interactions.

## Introduction

Plants are sessile organisms that have evolved a wealth of chemical and physical defense mechanisms to cope with environmental challenges. Among these, herbivores are important stress agents, exerting a strong selective pressure, which promoted the evolution of plant morphological and biochemical traits conferring protection. Spinescence, pubescence, sclerophylly and raphides are morphological adaptations protecting plants from herbivore attacks (Hanley *et al*., 2007), which are complemented by a high diversity of toxic/antinutritional molecules, such as alkaloids, phenols and proteinase inhibitors (Becerra, 2007). There is a large body of knowledge on these chemical defense barriers (War *et al*., 2012), while comparatively little is known about mechanisms based on the modulation of plant elements that strongly influence herbivore fitness, such as sodium (Cheeseman, 2013). Indeed, given the high importance of sodium in herbivores' diet, this element has been recently defined as the seventh macronutrient (Kaspari, 2020). Herbivores generally starve for sodium, since most plant species are highly efficient sodium excluders (Cheeseman, 2015). As a consequence, several ungulates search for sodium deposition in mud or urine of other animals (Welti *et al*., 2019), as well as on steep sides of dams (Biancardi & Minetti, 2017). Herbivore insects also show similar vital needs for sodium. Larvae of *Helicoverpa armigera* fed with suboptimal sodium levels have impaired growth, higher incidence of cannibalism and, when attaining the adult stage, lower flight speed (Xiao *et al*., 2010; Santiago‐Rosario *et al*., 2023). Moreover, for *Aphis glycines* (Eichele‐Nelson *et al*., 2018), it was found that as salinity increased, both population size and fecundity increased across electrical conductivity values ranging from 0.84 to 8.07 dS m^−1^ (Eichele‐Nelson *et al*., 2018). This was associated with longer survival and a more pronounced preference for high‐salinity plants (Eichele‐Nelson *et al*., 2018).

Sodium has important energetic, osmoregulatory and neurophysiological functions in animals (Filipiak & Filipiak, 2020; Santiago‐Rosario *et al*., 2025; Snell‐Rood *et al*., 2014), which suggests that plants can exclude sodium from their tissues as a strategy to contrast animal herbivory (Britto *et al*., 2021). In contrast, plants need sodium only in traces as a micronutrient. When exceeding optimal concentration, sodium excess is toxic to leaves and roots and it impairs several pathways of plant metabolism (Kronzucker *et al*., 2013). The vast majority of plants are indeed sodium‐excluding plants (glycophytes) over sodium accumulators (halophytes), which suggests that plants may exclude sodium from their tissues as a strategy to limit damages by herbivorous animals (Britto *et al*., 2021). A much higher sodium concentration in herbivores vs plant tissues (100‐ to 1000‐fold higher) further supports this hypothesis (Xiao *et al*., 2010). Along this line, Cheeseman (2015) has proposed that glycophytism could be a plant defense strategy against herbivores, particularly in environments where sodium is scarce. However, even though leaf sodium concentration and herbivore attraction are positively correlated (Kaspari, 2020), direct evidence unequivocally supporting the role of sodium exclusion as a strategy to reduce feeding damage remains largely elusive.

Here we contribute to address this research gap by the following experimental approaches: (1) assess whether sodium translocation occurs in response to insect attack; (2) evaluate the effect of sodium level in insect diet on growth, survival rate and molting; (3) compare the degree of leaf damage and larval feeding preference between leaves with high vs low sodium levels. The model systems used to test these hypotheses were tomato (*Solanum lycopersicum*) and the lepidopteran pest *Spodoptera littoralis* (Lepidoptera, Noctuidae).

## Materials and Methods

### Growth conditions and experimental design

The experiments were carried out in a 16‐m^2^ walk‐in grow‐room at the Department of Agricultural Sciences, University of Naples Federico II (latitude 40.814048, longitude 14.346258). Seeds of ‘San Marzano nano’ tomato (*Solanum lycopersicum* L.) were germinated in styrofoam trays filled with perlite and subirrigated with a ¼ strength modified Hoagland nutrient solution and were transplanted 20 d after sowing in 6 × 7 cm plastic baskets filled with perlite, floating in 2‐l buckets filled with nutrient solution. Each bucket was oxygenated with a small tube connected to a water oxygenator (Haquoss Turbolence 8400), with 100 W power and 140 l min^−1^ flow rate for a total of 3.5 l min^−1^ of air per bucket. The nutrient solution was a ½ strength for the first week and then a full strength modified Hoagland nutrient solution. The nutrient solution at its full strength consisted of 6.81 g l^−1^ of KH_2_PO_4_, 0.14 g l^−1^ of KNO_3_, 0.51 g l^−1^ of 5[Ca(NO_3_)_2_ • 2H_2_O] • NH_4_NO_3_, 1.18 g l^−1^ of MgSO_4_ and 0.03 g l^−1^ of micronutrients (Mugasol Mix, Mugavero, Termini Imerese, IT). Two nutrient solution treatments were prepared, differing in sodium concentration: the +Na treatment, corresponding to normal sodium levels, was obtained by adding 0.06 g l^−1^ of NaCl; the −Na treatment, corresponding to reduced sodium levels, was obtained by adding 0.06 g l^−1^ of CaCl₂. The use of CaCl₂ in the −Na treatment allowed balancing chloride content while ensuring only minimal differences in calcium concentration (< 0.5 mM). The pH of the solutions was kept between 5.5 and 6.0. To keep the sodium level at desired concentrations, Milli‐Q water was used for the preparation of the nutritive solution during all the growing stages. The average concentration of sodium that was present in nutrient solutions during the growth cycle was 1.67 mM for +Na solution and 0.34 mM for −Na solution with slight fluctuations between the nutrient solution changes (Supporting Information Fig. S1). The minimal sodium concentration in −Na solution was due to the traces of sodium in the phosphate fertilizer KH_2_PO_4_ used for the nutrient solution. The determination of sodium content in the nutrient solution has been performed with Laqua Twin Compact Water Quality Meter (HORIBA, Ltd, Kyoto, Japan) on 200 to 300 μl samples of nutrient solution per pot. Before use, the instrument has been calibrated to ensure the replicability of the measurements.

### Physiological traits and ion content

Ion content was analyzed, focusing on sodium as well as other relevant elements, including potassium, calcium and nitrate. Moreover, to evaluate the overall impact of different sodium regimes on plant performance, we assessed shoot and root biomass, leaf dry matter content, gas exchange parameters and photosystem II efficiency (*F*_v_/*F*_m_). Briefly, CO_2_ assimilation (μmol CO_2_ m^−2^ s^−1^), stomatal conductance (mol H_2_O m^−2^ s^−1^) and transpiration (mmol H_2_O m^−2^ s^−1^) were measured on 6‐wk‐old +Na and −Na tomato plants. The measurements have been done with a LI‐6400 (LI‐COR Biosciences, Lincoln, NE, USA) at ambient CO_2_ concentration (*c*. 400 μmol) and photosynthetic active radiation of 1000 μmol m^−2^ s^−1^. Measurements were performed from 10 am to 2 pm on a fully expanded leaf per plant. On the same plants, Chl fluorescence was measured using a FluorPen FP110 (PSI – Photon Systems Instruments, Drásov, Czech Republic). Measurements were carried out on the youngest fully expanded leaves. Before measurement, leaves were dark‐adapted for 30 min using the clips supplied by the manufacturer. The flash pulse intensity was set to 30% (measuring light pulses). These weak measuring pulses induce the minimum Chl fluorescence (F0 or Ft). The saturating pulse intensity was set to 10%, with 100% corresponding to *c*. 3000 μmol m^−2^ s^−1^. The saturating pulse induces maximum Chl fluorescence (*F*_m_). In dark‐adapted samples, the parameter Fv/Fm represents the maximum efficiency (quantum yield) of primary photochemistry in PSII.

Plants were separated into leaves, stems and roots, weighed to obtain fresh weight, then oven‐dried at 60°C until constant weight to determine dry biomass.

For the ion content determination, tissue sap was extracted by centrifuge 5425 R (Eppendorf) at 9391 ***g*** for 10 min using Eppendorf Corning® Costar® Spin‐X® centrifuge tube filters. The sap was analyzed using Laqua Twin Compact Water Quality Meter (HORIBA, Ltd).

### Glasshouse experiments with *S. littoralis* larvae

*Spodoptera littoralis* (Boisd.) larvae were obtained from a colony maintained at the Laboratory of Entomology ‘E. Tremblay’, Department of Agricultural Sciences, University of Naples Federico II. Insects were reared on an artificial diet (41.4 g l^−1^ wheat germ, 59.2 g l^−1^ brewer's yeast, 165 g l^−1^ corn meal, 5.9 g l^−1^ ascorbic acid, 1.53 g l^−1^ benzoic acid, 1.8 g l^−1^ methyl‐4‐hydroxybenzoate and 29.6 g l^−1^ agar) at 25 ± 1°C, 70 ± 5% RH and a 16 : 8 h light : dark photoperiod until pupation.

Day‐one fourth instar larvae were synchronized by selecting third instars close to molting (head capsule slippage visible) before the onset of scotophase and collecting newly molted larvae the following morning. These synchronized larvae were used for all *in vivo* assays, which were conducted in glasshouse conditions, using nine plants per treatment, each considered as an independent replicate.

The first experiment was designed to investigate whether larval feeding could induce sodium redistribution in +Na plants. To this end, larvae were placed on fully expanded leaves at the second, third, fourth and fifth nodes (two larvae per leaf), with each leaf enclosed in a tulle drawstring bag (10 × 6 cm) to confine the larvae to a single leaf. Leaves of control plants were also bagged, but without larvae. Leaves from both infested and control plants were collected at 3, 24, 48 and 72 h after the beginning of the assay (corresponding to leaves at the second, third, fourth and fifth nodes, respectively). At each sampling time, bags and larvae were removed, leaves were photographed, detached and immediately frozen in liquid nitrogen for RNA extraction. Leaf images were analyzed with ImageJ (U.S. National Institutes of Health, Bethesda, MD, USA) to quantify the damaged area. At the end of the experiment, 72 h post‐infestation, to evaluate overall sodium distribution in response to insect attack, roots, stems and leaves were separately collected and processed as described above. These analyses have been performed on nine biological replicates per treatment and the experiment has been replicated twice.

A second set of bioassays was performed using both +Na plants and −Na plants to test whether a different sodium availability could alter larval physiology and feeding behavior. Synchronized fourth instar larvae were individually weighed and placed on fully expanded leaves at the second, third and fourth nodes (two larvae per leaf), each leaf enclosed in a tulle drawstring bag as described above. Control plants (+Na and −Na plants) were bagged in the same way but without larvae. Larvae were allowed to feed for 24, 48 and 72 h. At these time points, larval growth, body weight and leaf consumption were quantified under the two nutritional regimes. At each time point and for each experimental condition, nine biological replicates (leaf and feeding larva) were collected to assess larval weight, leaf damage and plant gene expression. This experimental set‐up was replicated twice.

### RNA extraction, cDNA synthesis and qRT‐PCR

Total RNA from 100 mg of leaf tissue was extracted using RNeasy Plant Mini Kit (Qiagen) according to manufacturer's instructions. One microgram of RNA was reverse transcribed using QuantiTect Reverse Transcription Kit (Qiagen) according to the manufacturer's instructions. The complementary DNA was diluted 1 : 20 and 4.5 μl of the dilution were used in each reaction with 6.25 μl of PowerUp SYBR Green Master Mix (Applied Biosystems, Foster City, CA, USA) and 1.75 μl of primer mix (4.28 μM). The reaction was performed using 7900 HT Fast Real‐Time PCR System (Applied Biosystems). Cycling conditions were 10 min at 95°C, followed by 95°C for 15 s and 60°C for 1 min, 40 cycles. The *SlEF1α* (*Solyc06g005060*) was used as endogenous control and RNA extracted from plants grown in control condition as calibrator sample. Since leaves were collected from different nodes (second to fifth), the expression values are reported relative to the corresponding undamaged controls collected at the same sampling time to isolate the effects of herbivores feeding within the same leaf rank. Three biological replicates with three technical replicates were used and the relative expression of *SlHKT1;1* (*Solyc07g014690*), *SlHKT1;2* (*Solyc07g014680*; Héreil *et al*., 2024) and *SlSOS1* (*Solyc01g005020*) were calculated using the $2^{-\Delta \Delta C_{t}}$ method (Livak & Schmittgen, 2001). Primers used are listed in Table S1.

### Laboratory feeding bioassays

Groups of newly hatched *S. littoralis* larvae were initially reared in plastic boxes (30 × 40 × 15 cm) bottom‐lined with 50 ml of 1.5% agar (w/v) and fed with sub‐apical leaves excised from experimental tomato plants. Leaves were randomly collected starting from the second node of 6‐wk‐old plants and replaced daily. To avoid confounding effects due to wound‐induced defense responses, only leaves from plants left mechanically undisturbed for at least 3 d were used (Glauser *et al*., 2008; Yan *et al*., 2013).

Feeding assays were replicated twice and each independent experiment was carried out with 16 larvae (day 1 third instar) per experimental condition, synchronized as described above. Experimental larvae were individually weighed and transferred into multi‐well plastic rearing trays (RT32W; Frontier Agricultural Sciences, Newark, DE, USA) bottom‐lined with 1 ml of 1.5% agar (w/v) and closed with perforated lids (RTCV4; Frontier Agricultural Sciences); each larva was daily supplied with 4 cm^2^ leaf disks. Bioassays were carried out in an environmental chamber at 25 ± 1°C under a 16 : 8 h light : dark photoperiod. Each assay was replicated twice.

The following parameters were recorded: daily larval survival; larval weight on day 1 of the third, fourth, fifth and sixth instar, with an additional larval weight measurement on day 3 of the sixth instar (late sixth instar), just before pupation; pupal weight (on day 3 of the pupal stage) and pupal survival; time to adult emergence and adult survival.

### Dual‐choice laboratory feeding bioassays

Dual‐choice laboratory assays were conducted to assess larval feeding preferences. Each test was performed in a 12‐cm diameter plastic Petri dish containing 1 ml of 1.5% (w/v) agar to maintain leaf turgor. Two 5 cm^2^ tomato leaf disks, excised from +Na or −Na plants, were placed on the agar surface in diametrically opposite positions. A single third‐instar larva (day 1, synchronized as previously described) was released at the center of the dish and allowed to choose between the two leaf disks. Larval position and feeding activity were recorded at 1, 5, 24 and 48 h. All assays were run simultaneously at 25 ± 1°C under a 16 : 8 h light : dark photoperiod. Larvae that did not feed or consumed both disks in comparable amounts at the time points indicated above were considered unresponsive. Each choice test was carried out with 10 larvae and replicated twice.

### Statistical analysis

Group differences were evaluated with two‐tailed, unpaired *t*‐tests after verifying model assumptions. Normality was assessed with the Shapiro–Wilk test and homogeneity of variances with Levene's and Bartlett's tests. When equal variances were supported, Student's *t*‐test was used; otherwise, Welch's correction was applied. Survival of *S. littoralis* larvae and adults was analyzed by Kaplan–Meier estimation and compared using the log‐rank test. Choice‐assay outcomes were analyzed by χ^2^ goodness‐of‐fit tests, with expected frequencies defined by the corresponding control group. All analyses were performed in GraphPad Prism (GraphPad Software, San Diego, CA, USA).

## Results

### Sodium translocation in tomato plants induced by larval feeding

**Fig. 1:**
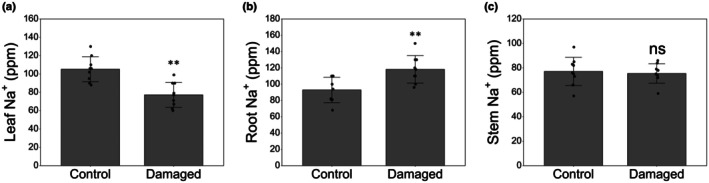
Sodium concentration in leaves (a), roots (b) and stem (c) in undamaged control *Solanum lycopersicum* plants and in plants damaged by *Spodoptera littoralis* larvae, 72 h after larvae were placed on leaves. Data reported are means ±SD of nine replicates. Asterisks indicate significant differences between the two treatments according to Student's *t*‐test (**, *P* < 0.01; ns, not significant).

To test the hypothesis that plants regulate sodium translocation in response to insect attack, the ion concentration in leaves, stems and roots of plants grown under standard sodium nutrition (+Na) was measured 72 h after the onset of larval feeding. The sodium concentration in the leaf was 27.2% lower in damaged plants (Fig. 1a), whereas root sodium concentration was 30.7% higher in damaged plants (Fig. 1b). Sodium stem concentration did not show significant differences between the two treatments (Fig. 1c).

Moreover, the total plant sodium content, obtained as the sum of the products obtained by multiplying tissue sodium concentration by the biomass of leaves, stem and roots, was 24.5% lower in damaged vs undamaged plants (Fig. S2). Therefore, sodium reallocation from the leaf to the root was likely associated with an overall reduction of sodium uptake. On the contrary, the concentration of other ions (K^+^, Ca^2+^ and NO_3_^−^) was not affected by larval feeding (Table S2).

**Fig. 2:**
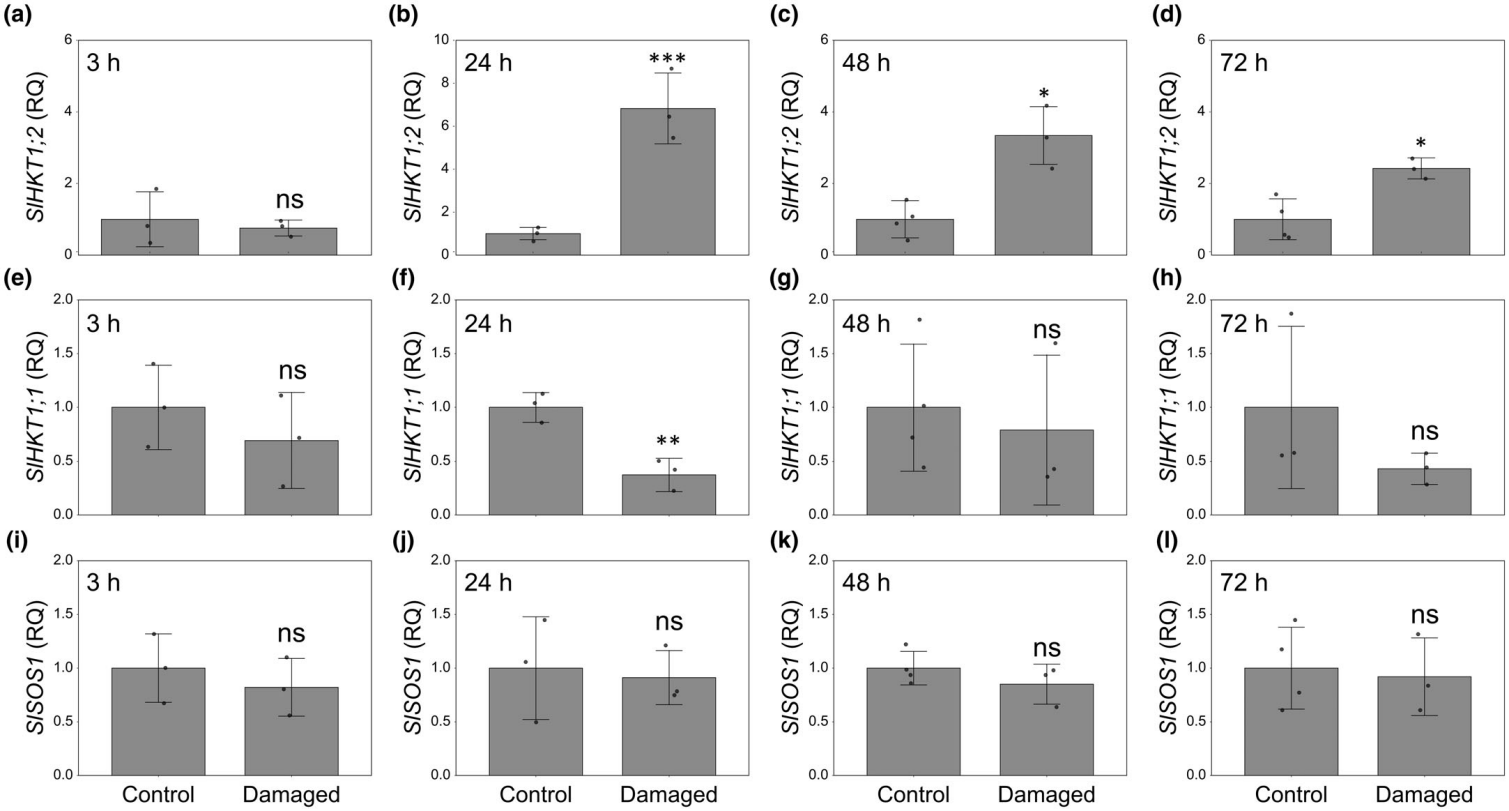
Expression analysis of *SlHKT1;2* (a–d), *SlHKT1.1* (e–h) and *SlSOS1* (i–l) in *Solanum lycopersicum* leaves of +Na plants, 3, 24, 48 and 72 h after infestation by *Spodoptera littoralis* larvae and in synchronous undamaged control +Na plants. Data were normalized using RNA from control plants. Data reported are means ±SD of three biological replicates. Asterisks indicate significant differences between the two treatments according to Student's *t*‐test (*, *P* < 0.05; **, *P* < 0.01; ***, *P* < 0.001; ns, not significant).

The evidence obtained from sodium quantification prompted us to analyze the expression profiles of the main genes involved in sodium transport and homeostasis. In particular, we analyzed the tomato genes encoding the sodium‐selective plasma membrane transporters *SlHKT1;1* and *SlHKT1;2* (High‐affinity Potassium Transporter from class I). These two genes have been proposed to play a role in Na^+^ recirculation in plants, with *SlHKT1;1* responsible for xylem loading and phloem unloading, while *SlHKT1;2* responsible for xylem unloading and phloem loading (Asins *et al*., 2022). The expression of *SlHKT1;2* was not different between control and damaged plants at 3 h post‐infestation (Fig. 2a), yet it was 6‐, 3‐ and 2.4‐fold higher in damaged leaves than in the corresponding undamaged controls at 24, 48 and 72 h of feeding activity, respectively (Fig. 2b–d). The expression of *SlHKT1;1* was significantly reduced (fold change: 0.36) only at 24 h post‐infestation (Fig. 2f). Interestingly, the expression of *SlSOS1* (SALT OVERLY SENSITIVE 1), an Na^+^/H^+^ plasma membrane antiporter which mediates Na^+^ efflux and xylem loading (Shi *et al*., 2002), did not show significant differences under larval feeding (Fig. 2i,l).

These findings suggest that larval feeding leads to a redistribution of sodium within the plant, with reduced levels in the damaged leaves and increased accumulation in the roots. This physiological shift is consistent with the transcriptional modulation of specific sodium transporters, providing coherent evidence that feeding damage by a chewing insect (in this case *S. littoralis*) results in sodium reallocation from infested (leaves) to non‐infested (roots) tissues.

### Effects of different sodium levels on plants

**Fig. 3:**
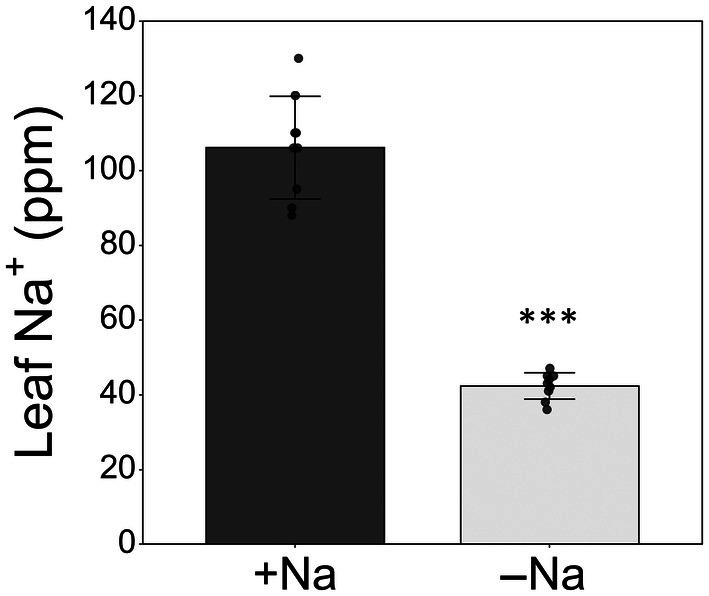
Leaf sodium content of *Solanum lycopersicum* plants grown with nutrient solution under +Na and −Na conditions. Data reported are means ±SD of nine replicates. Asterisks indicate significant differences between the two treatments according to Student's *t*‐test (***, *P* < 0.001).

To evaluate whether different concentrations of sodium affected the overall plant physiology, we measured biomass accumulation and physiological traits in plants grown under different sodium regimes (−Na and +Na). Plants grown under +Na showed 2.5‐fold higher leaf sodium content compared to −Na plants (Fig. 3). The gas exchange parameters and photosystem II efficiency (*F*_v_/*F*_m_) remained unaffected by the different nutrient solutions (Fig. S3). Similarly, shoot and root biomass did not differ between −Na and +Na plants, nor did the dry matter content of the leaves (Fig. S4). To check whether other relevant nutrients for plant growth and/or ion homeostasis were affected by reduced Na levels, the concentrations of potassium, calcium and nitrate were also measured. No differences were found for these ions between −Na plants and +Na plants (Table S3). To assess if the modulation of sodium levels in plants could unexpectedly induce defense responses affecting insects feeding on them, we checked the expression level of key genes related to activation of plant barriers against chewing insects (Lee *et al*., 1986; Orozco‐Cardenas *et al*., 1993; Peña‐Cortés *et al*., 1995; Ryan & Pearce, 1998). The expression of *SlProsys* (encoding pro‐systemin, a key protein in tomato plants that serves as a precursor to the 18‐amino acid peptide signaling molecule systemin), *SlPinI* (a serine‐type endopeptidase inhibitor) and *SlPinII* (a Proteinase Inhibitor II) did not show significant differences between undamaged +Na and −Na plants (Fig. S5). Collectively, these results indicate that the most relevant change recorded in −Na plants was the reduced Na level compared to +Na plants.

### Effects of different plant sodium levels on insects

**Fig. 4:**
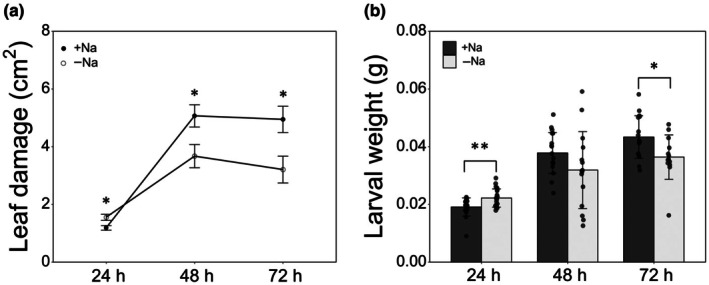
Damaged area of *Solanum lycopersicum* leaves (a) and larval weight (b) from +Na plants and *−*Na plants, 24, 48 and 72 h after infestation by *Spodoptera littoralis* larvae (*n* = 18). Data reported are means ±SE (a) and ±SD (b) of 18 replicates. Asterisk indicates significant differences according to Student's *t*‐test (*, *P* < 0.05; **, *P* < 0.01).

In glasshouse experiments, 24 h after the infestation by *S. littoralis* larvae, the damaged area on leaves from −Na plants was significantly higher than that on leaves from +Na plants (+30.6%). The reverse was observed at 48 and 72 h after larval infestation, with a significant difference of the damaged area, which was 38 and 54% higher in +Na plants compared to −Na plants, respectively (Fig. 4a). Regarding the larval growth on plants from the two groups (+Na and −Na), the weight registered over time mirrored the trend of leaf damage. Indeed, larval weight was significantly higher in −Na plants compared to +Na plants only at 24 h post‐infestation, while at the following time points the reverse was observed, with a significant weight increase registered for +Na plants at 72 h after the onset of larval feeding (Fig. 4b). It is interesting to note that the expression of key defense‐related genes (*SlProsys*, *SlPin* and *SlPinII*) was, in general, significantly upregulated upon larval feeding both in +Na and −Na plants, and likely positively associated with the feeding damage, as detailed, for each gene, in Figs S6–S8. The total absence of larval mortality during this short experiment indicates that any plant defense barriers, either constitutive or induced by plant treatment and/or larval feeding, do not exert an acute toxicity, but rather determine an alteration of the feeding behavior and growth patterns.

**Fig. 5:**
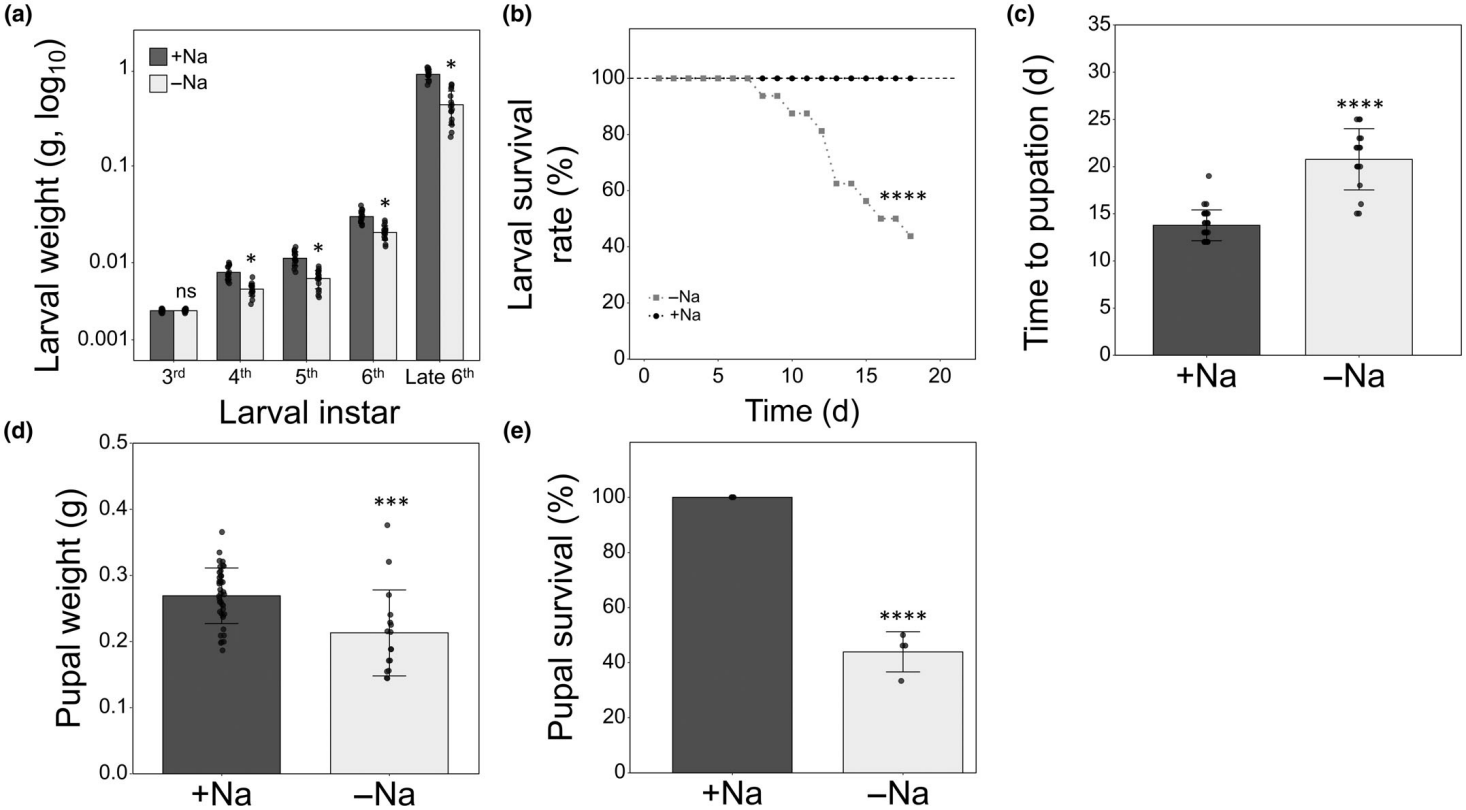
Effect of −Na *Solanum lycopersicum* plants on *Spodoptera littoralis* body weight across instars (a), survival rate (b), time to pupation (c), pupal weight (d) and pupal survival (e). Asterisks denote significant differences between groups according to: (a) multiple *t*‐tests (*, *P* < 0.05; *n* = 32); (b) log‐rank Mantel‐Cox test (****, *P* < 0.0005; *n* = 32); (c) Kolmogorov–Smirnov test (****, *P* < 0.0001; N_+Na_ = 32 and N_−Na_ = 16); (d) unpaired *t*‐test with Welch's correction (***, *P* < 0.0005; N_+Na_ = 32 and N_−Na_ = 16); (e) Fisher's exact test (****, *P* < 0.0001; N_+Na_ = 32 and N_−Na_ = 16). Error bars indicate ±SD.

To determine whether these alterations could bear fitness consequences, we performed laboratory bioassays tracking experimental insects throughout development. Feeding on leaves from −Na plants reduced larval growth and survival relative to +Na plants, as reflected by a significant decrease in body weight across development, becoming significant from the fourth instar to late sixth instar (Fig. 5a), and with survival declining to *c*. 40% by the end of the assay (Fig. 5b). Surviving larvae on −Na plants took longer to pupate (Fig. 5c) and attained a lower pupal weight (Fig. 5d). These pupal deficits were associated with *c*.60% pupal mortality, whereas 100% of pupae from the +Na treatment survived until adult emergence (Fig. 5e).

**Fig. 6:**
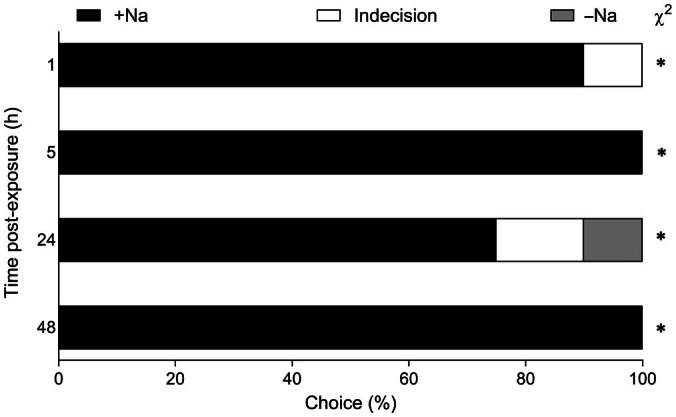
Choice trial between leaves of *Solanum lycopersicum* plants grown under +Na and −Na conditions of single *Spodoptera littoralis* larvae (*n* = 20). Asterisk indicates significant differences in the χ^2^ test (*, *P* < 0.05).

To assess if the altered feeding behavior was also accompanied by changes in food preference, we performed a dual‐choice bioassay, which revealed a significant and sustained larval preference for +Na leaves across all time points assessed (Fig. 6).

## Discussion

Plants have evolved complex mechanisms for sodium exclusion and reallocation that have led these organisms to reduce sodium concentration in their tissues, notwithstanding its ubiquitous presence in the environment (Wu, 2018). Excluding a small number of halophytes, most plant species do not tolerate high levels of sodium in their tissues (Cheeseman, 2015). Salt stress caused by NaCl is indeed one of the main abiotic constraints for plant growth and productivity. Ion transporters, such as SOS1, HKTs and NHXs (Na^+^/H^+^ antiporters), play complementary roles in salt tolerance by mediating Na^+^ extrusion, retrieval and vacuolar sequestration (Olías *et al*., 2009; Almeida *et al*., 2017; Ali *et al*., 2023; Cavusoglu *et al*., 2023), and have been among the first genetic targets for improving plant salt stress tolerance (Wu, 2018). It has also been hypothesized that, as in the case of secondary metabolites, plants can modulate their ionome to reduce their palatability and/or nutritional value for herbivores feeding, thus reducing their infestation level (Kaspari, 2020). Our results demonstrate that upon larval feeding, the leaf Na^+^ level was reduced compared to control plants (Fig. 1a). By contrast, Na^+^ root concentration was found to be higher in damaged plants, likely indicating a translocation of Na^+^ from the leaf to the root which must occur via phloem transport (Fig. 1b). The concentration of other ions (K^+^, Ca^2+^ and NO_3_^−^) did not show significant changes following larval feeding (Table S2), which may suggest the activation of a sodium‐specific mechanism. This hypothesis is further supported by a significant upregulation, triggered by larval feeding, of *SlHKT1;2* (Fig. 2b–d), a gene involved in removing Na^+^ from the xylem sap, preventing its toxic accumulation in leaves and shoots. Previous studies have reported *SlHKT1;2* expression in the vascular tissues of leaves and roots, with localization in both xylem and phloem cells (Almeida *et al*., 2014; Jaime‐Pérez *et al*., 2017). Besides its primary role in unloading sodium from the xylem to protect photosynthetic tissues, in the wild species *S. cheesmaniae* was detected *Scheme KT1;2* expression in the shoot phloem, where it likely loads Na^+^ into phloem to facilitate transport toward the roots (Romero‐Aranda *et al*., 2021; Asins *et al*., 2022). Silencing of *SlHKT1;2* causes over‐accumulation of sodium in leaves of both *S. lycopersicum* and *S. cheesmanie* under salt stress, confirming a central role of this gene in sodium recirculation from shoots to roots (Jaime‐Pérez *et al*., 2017; Romero‐Aranda *et al*., 2020).

**Fig. 7:**
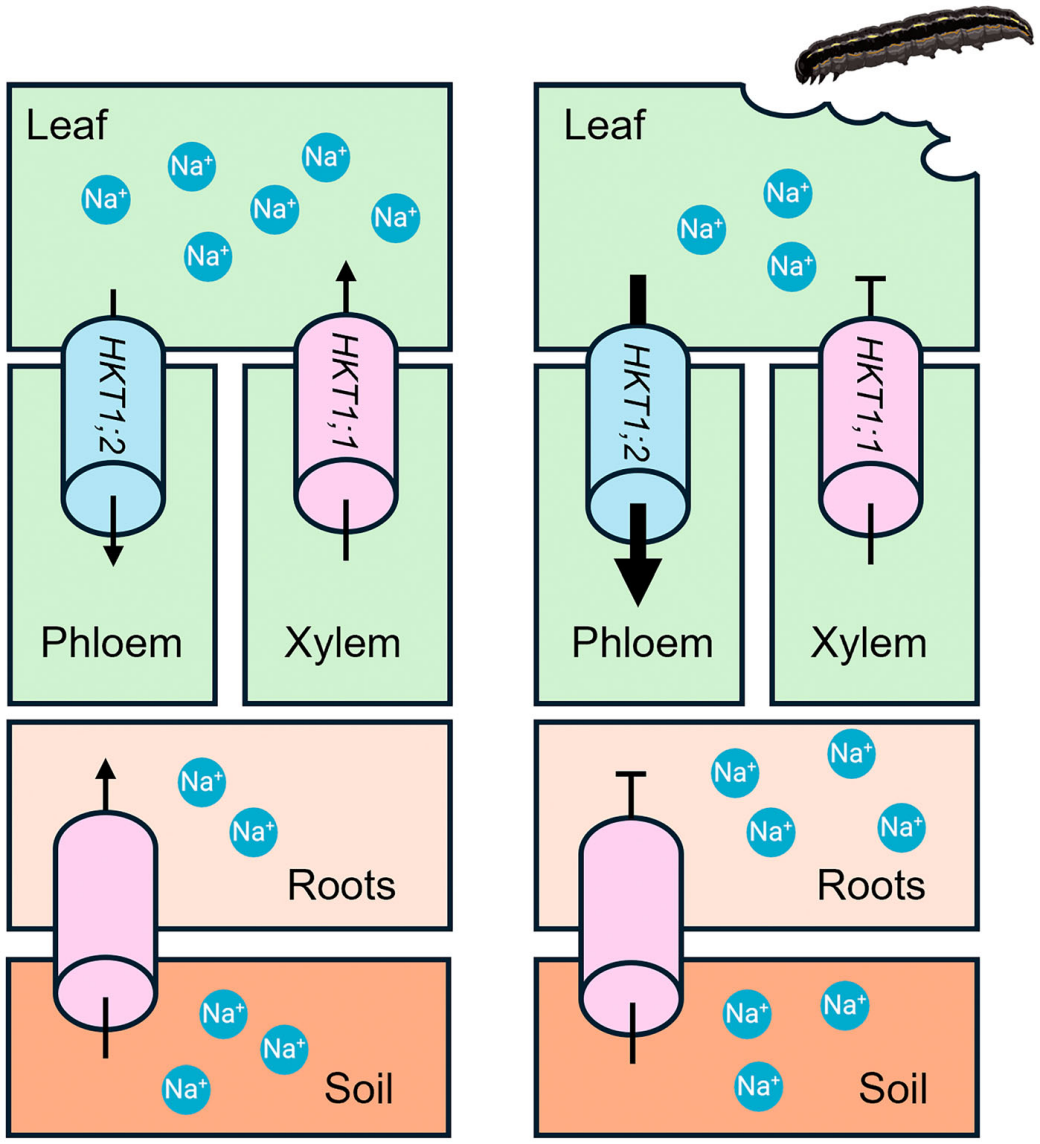
Proposed model of sodium reallocation following *Spodoptera littoralis* attack on leaves of *Solanum lycopersicum*. *HKT1;1* and *HKT1;2* play, among others, an important role in the distribution in plants of sodium absorbed by roots (left column). Larval damage to leaves (right column) causes downregulation of *HKT1;1*, which reduces the loading of sodium in the xylem and contributes to maintaining low leaf sodium levels. Concurrently, larval attack upregulates *HKT1;2*, which favors the phloem load of sodium and its translocation to the roots. A coordinated function of these genes mediates the redistribution of sodium throughout the plant, causing lower sodium concentrations in the leaves and higher sodium concentrations in the roots of damaged vs undamaged plants. Since larval damage determines a reduction of the total sodium in plants, it is reasonable to hypothesize that this challenge interferes with sodium uptake by roots by targeting ion transport systems that remain to be identified.

The contrasting transcriptional responses observed following herbivore attack, namely *SlHKT1;1* downregulation and *SlHKT1;2* upregulation, may be associated with reduced sodium loading into the xylem and enhanced sodium redistribution toward the roots via the phloem. These results are graphically represented in Fig. 7.

The scenario depicted above indicates that feeding by *S. littoralis* larvae on tomato plants activates a mechanism of sodium translocation, moving this ion from the herbivore feeding site (leaf) to a different plant organ (roots), thus reducing the nutritional value/palatability of the leaf. This aspect is of particular interest, since the activation of basipetal sodium translocation in pest‐damaged plants may provide a further protection barrier poorly recognized so far.

The protection conferred by this physiological response induced by insect feeding is well supported by our results, showing that larvae of *S. littoralis* feeding on leaves from −Na tomato plants, which grew on a reduced Na concentration (Fig. 3) that yet did not impair plant growth and other fundamental physiological responses, showed lower body weight (Figs 4b, 5a), lower larval and pupal survival (Fig. 5b,e), pupation delay (Fig. 5c) and reduced pupal weight (Fig. 5d). This is in line with a few literature reports on the impact of sodium on insect physiology and development. Herbivore insects feeding on a low sodium plant substrate showed reduced growth rates and other symptoms such as cannibalism and lower flight speed (Xiao *et al*., 2010). Moreover, plants living in environments where sodium was not a limiting factor showed higher levels of herbivore damage compared to plants living in a sodium‐poor environment (Prather *et al*., 2018). Similarly, Welti & Kaspari (2021) showed that sodium addition in different grasslands increased the occurrence of herbivory and fungal damage on plants, supporting the centrality of sodium in the food web where plants are primary sources of nutrients.

Collectively, our results shed light on a complex molecular network regulating sodium reallocation in tomato plants in response to insect feeding. This is a novel defense barrier unrecognized so far, likely of wide occurrence in other insect‐plant systems, which deserves further exploration.

Sodium is critical for insect development and normal physiology (Dow, 2017), and sodium deficiency in insects leads to enhanced scavenging for this ion (Wang *et al*., 2025). Since sodium is not uniformly distributed in the environment, this has driven the evolution of its active search by animals (Hurley & Johnson, 2015). Our results showed that leaf damage by *S. littoralis* larvae was lower in −Na plants compared to +Na plants (Fig. 4a), which was associated with a reduced food intake, starting 48 h after infestation (Fig. 4b). It is worth noting that larvae feeding on −Na plants caused higher damage during the first 24 h from the beginning of infestation (Fig. 4a), and their weight was higher compared to control larvae (Fig. 4b), suggesting that larvae feeding on −Na plants may perceive a sodium need that may trigger some kind of compensatory feeding, which is later reversed by physiological changes in feeding insects, resulting in altered control of food intake by the nervous system and/or nutrient absorption by the gut, both influenced by ion balance. Future studies will have to focus on the mechanisms underlying these physiological changes. Whatever the mechanism, the available data allow us to hypothesize that the presence of toxic secondary metabolites, induced by sodium treatment and/or reinforced by larval feeding, can be reasonably ruled out and that the sodium concentration of the plant tissues can be responsible for the observed impact on insects, by interfering with food uptake and use. It will be necessary to unravel if and how the changes in the ion balance both in the insect gut and body cavity can induce a physiological redirection inducing the observed alterations.

This effect is further reinforced by the feeding behavior of *S. littoralis* larvae, which showed a strong preference for +Na leaves over −Na leaves (Fig. 6). Insects ability to discriminate between different sodium levels is well documented (Mitchell *et al*., 2019). Sodium sensors have been thoroughly studied in hematophagous insects for prey choice (Pontes *et al*., 2017; Sarkissian *et al*., 2022), and for fruit choice in *Drosophila melanogaster* (Dweck *et al*., 2022). Other examples of sodium preference in insects are present in the literature. Spraying a NaCl solution on soybean plants increased the food‐touching behavior of adults belonging to different pentatomid species, thus enhancing the efficacy of the insecticide treatment (Corso & Gazzoni, 1998). Different plant species enriched with sodium resulted more attractive for pollinators (Finkelstein *et al*., 2022). Similarly, different leaf cutter ants are attracted by leaves with higher sodium concentrations (O'Donnell *et al*., 2010; Chavarria Pizarro *et al*., 2012). The available experimental evidence clearly indicates the importance of sodium in the insect diet and in the regulation of its feeding behavior. This offers the opportunity to manipulate host plant selection by pest insects by modulating sodium concentration in plant leaves. Further work is required to assess the feasibility of this approach under realistic field conditions.

### Conclusions

Sodium plays a multifaceted role in plant biology and appears to be of particular importance in the context of plant–herbivore interactions. The asymmetric requirements of plants and herbivores make sodium a key‐factor in their coevolution. Our results demonstrate the existence of a new plant defense pathway based on sodium translocation activated in response to insect infestation. This finding opens new perspectives on the study of the crosstalk between abiotic (e.g. salt stress) and biotic (e.g. herbivore attacks) stress responses in plants and provide novel insights into the coevolutionary dynamics between plants and herbivores. These results may also imply that molecular responses commonly considered as part of plant tolerance to salt stress, such as Na translocation/redistribution/compartmentalization, may have primarily developed as a defense strategy toward herbivores, among which insects play a crucial role, being the most abundant creatures on Earth, with a long and intense evolutionary arms race with plants. This interesting novelty has also significant applied implications, paving the way toward the development of plant protection strategies based on the modulation of this defense pathway, which has been largely overlooked so far.

## Competing interests

None declared.

## Author contributions

VC, IDL, AB, MVM, A. Maggio and FP planned and designed the research. PP, MC, A. Marciano, CR, NP, GJ, MGDL and MC performed experiments. VC, IDL, GJ, MGDL, PP and CR analyzed data. VC, IDL, PP, A. Maggio, FP and MVM wrote the manuscript. VC and IDL contributed equally to this work.

## Disclaimer

The New Phytologist Foundation remains neutral with regard to jurisdictional claims in maps and in any institutional affiliations.

## Supporting information


**Fig. S1** Sodium concentration in the nutrient solution expressed in ppm during the growth cycle *of Solanum lycopersicum plants* in +Na and −Na treatments.
**Fig. S2** Total sodium content and its distribution in leaves, stem *and* root, in *Solanum lycopersicum* undamaged control +Na plants and in +Na plants damaged by *Spodoptera littoralis* larvae.
**Fig. S3** Plant physiological traits of *Solanum lycopersicum* plants grown under +Na and −Na treatments.
**Fig. S4** Plant biometric traits of *Solanum lycopersicum* plants grown under +Na and −Na treatments.
**Fig. S5** Expression analysis of SlPinI, SlPinII and SlProsys in *Solanum lycopersicum* leaves of undamaged +Na and −Na plants.
**Fig. S6** Expression analysis of SlProSys in *Solanum lycopersicum* leaves of +Na and −Na plants at 24, 48 and 72 h after *Spodoptera littoralis* infestation.
**Fig. S7** Expression analysis of SlPinI in *Solanum lycopersicum* leaves of +Na and −Na plants at 24, 48 and 72 h after *Spodoptera littoralis* infestation.
**Fig. S8** Expression analysis of SlPinII in *Solanum lycopersicum* leaves of +Na and −Na plants at 24, 48 and 72 h after *Spodoptera littoralis* infestation.
**Table S1** Primers used in this study.
**Table S2** Ion content of *Solanum lycopersicum* leaves grown under +Na and subjected to larvae attack.
**Table S3** Ion content of *Solanum lycopersicum* leaves grown under +Na vs −Na.
Please note: Wiley is not responsible for the content or functionality of any Supporting Information supplied by the authors. Any queries (other than missing material) should be directed to the *New Phytologist* Central Office.

## Data Availability

The data that support the findings of this study are openly available in Zenodo at 10.5281/zenodo.19005807.

## References

[nph71150-bib-0001] Ali A , Petrov V , Yun D‐J , Gechev T . 2023. Revisiting plant salt tolerance: novel components of the SOS pathway. Trends in Plant Science 28: 1060–1069. 37117077 10.1016/j.tplants.2023.04.003

[nph71150-bib-0002] Almeida DM , Oliveira MM , Saibo NJM . 2017. Regulation of Na^+^ and K^+^ homeostasis in plants: towards improved salt stress tolerance in crop plants. Genetics and Molecular Biology 40: 326–345. 28350038 10.1590/1678-4685-GMB-2016-0106 PMC5452131

[nph71150-bib-0003] Almeida P , De Boer G‐J , De Boer AH . 2014. Differences in shoot Na^+^ accumulation between two tomato species are due to differences in ion affinity of *HKT1;2* . Journal of Plant Physiology 171: 438–447. 24594396 10.1016/j.jplph.2013.12.001

[nph71150-bib-0004] Asins MJ , Romero‐Aranda MR , Espinosa J , González‐Fernández P , Jaime‐Fernández E , Traverso JA , Carbonell EA , Belver A . 2022. *HKT1;1* and *HKT1;2* Na^+^ transporters from *Solanum galapagense* play different roles in the plant Na^+^ distribution under salinity. International Journal of Molecular Sciences 23: 5130. 35563521 10.3390/ijms23095130 PMC9103179

[nph71150-bib-0005] Becerra JX . 2007. The impact of herbivore–plant coevolution on plant community structure. Proceedings of the National Academy of Sciences, USA 104: 7483–7488. 10.1073/pnas.0608253104 PMC1855276 17456606

[nph71150-bib-0006] Biancardi C , Minetti A . 2017. Gradient limits and safety factor of *Alpine ibex* locomotion. Hystrix, the Italian Journal of Mammalogy 28: 56–60.

[nph71150-bib-0007] Britto DT , Coskun D , Kronzucker HJ . 2021. Potassium physiology from Archean to Holocene: a higher‐plant perspective. Journal of Plant Physiology 262: 153432. 34034042 10.1016/j.jplph.2021.153432

[nph71150-bib-0008] Cavusoglu E , Sari U , Tiryaki I . 2023. Genome‐wide identification and expression analysis of Na^+^/H^+^ antiporter (*NHX*) genes in tomato under salt stress. Plant Direct 7: e543. 37965196 10.1002/pld3.543 PMC10641485

[nph71150-bib-0009] Chavarria Pizarro L , Mccreery HF , Lawson SP , Winston ME , O'Donnell S . 2012. Sodium‐specific foraging by leafcutter ant workers (*Atta cephalotes*, Hymenoptera: Formicidae). Ecological Entomology 37: 435–438.

[nph71150-bib-0010] Cheeseman JM . 2013. The integration of activity in saline environments: problems and perspectives. Functional Plant Biology 40: 759–774. 32481149 10.1071/FP12285

[nph71150-bib-0011] Cheeseman JM . 2015. The evolution of halophytes, glycophytes and crops, and its implications for food security under saline conditions. New Phytologist 206: 557–570. 25495078 10.1111/nph.13217

[nph71150-bib-0012] Corso IC , Gazzoni CL . 1998. An insecticide enhancer for controlling pentatomids on soybeans. Pesquisa Agropecuária Brasileira 33: 1563–1571.

[nph71150-bib-0013] Dow JA . 2017. The essential roles of metal ions in insect homeostasis and physiology. Current Opinion in Insect Science 23: 43–50. 29129281 10.1016/j.cois.2017.07.001

[nph71150-bib-0014] Dweck HKM , Talross GJS , Luo Y , Ebrahim SAM , Carlson JR . 2022. Ir56b is an atypical ionotropic receptor that underlies appetitive salt response in *Drosophila* . Current Biology 32: 1776–1787.e4. 35294865 10.1016/j.cub.2022.02.063 PMC9050924

[nph71150-bib-0015] Eichele‐Nelson J , DeSutter T , Wick AF , Harmon EL , Harmon JP . 2018. Salinity improves performance and alters distribution of soybean aphids. Environmental Entomology 47: 875–880. 29800282 10.1093/ee/nvy072

[nph71150-bib-0016] Filipiak ZM , Filipiak M . 2020. The scarcity of specific nutrients in wild bee larval food negatively influences certain life history traits. Biology‐Basel 9: 462. 33322450 10.3390/biology9120462 PMC7764569

[nph71150-bib-0017] Finkelstein CJ , CaraDonna PJ , Gruver A , Welti EAR , Kaspari M , Sanders NJ . 2022. Sodium‐enriched floral nectar increases pollinator visitation rate and diversity. Biology Letters 18: 20220016. 35232272 10.1098/rsbl.2022.0016 PMC8889166

[nph71150-bib-0018] Glauser G , Grata E , Dubugnon L , Rudaz S , Farmer EE , Wolfender J‐L . 2008. Spatial and temporal dynamics of jasmonate synthesis and accumulation in Arabidopsis in response to wounding. Journal of Biological Chemistry 283: 16400–16407. 18400744 10.1074/jbc.M801760200

[nph71150-bib-0019] Hanley ME , Lamont BB , Fairbanks MM , Rafferty CM . 2007. Plant structural traits and their role in anti‐herbivore defence. Perspectives in Plant Ecology, Evolution and Systematics 8: 157–178.

[nph71150-bib-0020] Héreil A , Guillaume M , Duboscq R , Carretero Y , Pelpoir E , Bitton F , Giraud C , Karlova R , Testerink C , Stevens R *et al*. 2024. Characterisation of a major QTL for sodium accumulation in tomato grown in high salinity. Plant, Cell & Environment 47: 5089–5103. 10.1111/pce.15082 39148196

[nph71150-bib-0021] Hurley SW , Johnson AK . 2015. The biopsychology of salt hunger and sodium deficiency. Pflugers Archiv: European Journal of Physiology 467: 445–456. 25572931 10.1007/s00424-014-1676-y PMC4433288

[nph71150-bib-0022] Jaime‐Pérez N , Pineda B , García‐Sogo B , Atares A , Athman A , Byrt CS , Olías R , Asins MJ , Gilliham M , Moreno V *et al*. 2017. The sodium transporter encoded by the *HKT1;2* gene modulates sodium/potassium homeostasis in tomato shoots under salinity. Plant, Cell & Environment 40: 658–671. 10.1111/pce.12883 27987209

[nph71150-bib-0023] Kaspari M . 2020. The seventh macronutrient: how sodium shortfall ramifies through populations, food webs and ecosystems. Ecology Letters 23: 1153–1168. 32380580 10.1111/ele.13517

[nph71150-bib-0024] Kronzucker HJ , Coskun D , Schulze LM , Wong JR , Britto DT . 2013. Sodium as nutrient and toxicant. Plant and Soil 369: 1–23.

[nph71150-bib-0025] Lee JS , Brown WE , Graham JS , Pearce G , Fox EA , Dreher TW , Ahern KG , Pearson GD , Ryan CA . 1986. Molecular characterization and phylogenetic studies of a wound‐inducible proteinase inhibitor I gene in *Lycopersicon* species. Proceedings of the National Academy of Sciences, USA 83: 7277–7281. 10.1073/pnas.83.19.7277 PMC386699 3463966

[nph71150-bib-0026] Livak KJ , Schmittgen TD . 2001. Analysis of relative gene expression data using real‐time quantitative PCR and the 2^−ΔΔ*C*T^ method. Methods 25: 402–408. 11846609 10.1006/meth.2001.1262

[nph71150-bib-0027] Mitchell TS , Shephard AM , Kalinowski CR , Kobiela ME , Snell‐Rood EC . 2019. Butterflies do not alter oviposition or larval foraging in response to anthropogenic increases in sodium. Animal Behaviour 154: 121–129.

[nph71150-bib-0028] O'Donnell S , García‐C JM , Beard J , Chiwocha T , Lewis D , Liu C , Phillips H , Williams T . 2010. Leaf cutter ants (*Atta cephalotes*) harvest baits offering sodium chloride rewards. Insectes Sociaux 57: 205–208.

[nph71150-bib-0029] Olías R , Eljakaoui Z , Pardo JM , Belver A . 2009. The Na^+^/H^+^ exchanger *SOS1* controls extrusion and distribution of Na^+^ in tomato plants under salinity conditions. Plant Signaling & Behavior 4: 973–976. 19826225 10.4161/psb.4.10.9679 PMC2801365

[nph71150-bib-0030] Orozco‐Cardenas M , McGurl B , Ryan CA . 1993. Expression of an antisense prosystemin gene in tomato plants reduces resistance toward *Manduca sexta* larvae. Proceedings of the National Academy of Sciences, USA 90: 8273–8276. 10.1073/pnas.90.17.8273 PMC47331 11607423

[nph71150-bib-0031] Peña‐Cortés H , Fisahn J , Willmitzer L . 1995. Signals involved in wound‐induced proteinase inhibitor II gene expression in tomato and potato plants. Proceedings of the National Academy of Sciences, USA 92: 4106–4113. 10.1073/pnas.92.10.4106 PMC41894 11607535

[nph71150-bib-0032] Pontes G , Pereira MH , Barrozo RB . 2017. Salt controls feeding decisions in a blood‐sucking insect. Journal of Insect Physiology 98: 93–100. 27989677 10.1016/j.jinsphys.2016.12.002

[nph71150-bib-0033] Prather CM , Laws AN , Cuellar JF , Reihart RW , Gawkins KM , Pennings SC . 2018. Seeking salt: herbivorous prairie insects can be co‐limited by macronutrients and sodium. Ecology Letters 21: 1467–1476. 30039540 10.1111/ele.13127

[nph71150-bib-0034] Romero‐Aranda MR , Espinosa J , González‐Fernández P , Jaime‐Fernández E , Traverso JÁ , Asins MJ , Belver A . 2021. Role of Na^+^ transporters *HKT1;1* and *HKT1;2* in tomato salt tolerance. I. Function loss of *cheesmaniae* alleles in roots and aerial parts. Plant Physiology and Biochemistry 168: 282–293. 34673319 10.1016/j.plaphy.2021.10.018

[nph71150-bib-0035] Romero‐Aranda MR , González‐Fernández P , Pérez‐Tienda JR , López‐Diaz MR , Espinosa J , Granum E , Traverso JÁ , Pineda B , Garcia‐Sogo B , Moreno V *et al*. 2020. Na^+^ transporter *HKT1;2* reduces flower Na^+^ content and considerably mitigates the decline in tomato fruit yields under saline conditions. Plant Physiology and Biochemistry 154: 341–352. 32604062 10.1016/j.plaphy.2020.05.012

[nph71150-bib-0036] Ryan CA , Pearce G . 1998. SYSTEMIN: a polypeptide signal for plant defensive genes. Annual Review of Cell and Developmental Biology 14: 1–17. 10.1146/annurev.cellbio.14.1.1 9891776

[nph71150-bib-0037] Santiago‐Rosario LY , Salgado AL , Paredes‐Burneo D , Harms KE . 2023. Low sodium availability in hydroponically manipulated host plants promotes cannibalism in a lepidopteran herbivore. Scientific Reports 13: 20822. 38012267 10.1038/s41598-023-48000-z PMC10682487

[nph71150-bib-0048] Santiago‐Rosario LY , Shephard AM , Snell‐Rood E , Herrmann AD , Harms KE . 2025. Butterfly species vary in sex‐specific sodium accumulation from larval diets. Ecological Entomology 50: 228–234.

[nph71150-bib-0038] Sarkissian T , Mazzio C , Garrity PA . 2022. Salt sensing: a new receptor for an ancient taste. Current Biology 32: R373–R375. 35472427 10.1016/j.cub.2022.01.021

[nph71150-bib-0039] Shi H , Quintero FJ , Pardo JM , Zhu J‐K . 2002. The putative plasma membrane Na^+^ /H^+^ antiporter SOS1 controls long‐distance Na^+^ transport in plants. Plant Cell 14: 465–477. 11884687 10.1105/tpc.010371 PMC152925

[nph71150-bib-0040] Snell‐Rood EC , Espeset A , Boser CJ , White WA , Smykalski R . 2014. Anthropogenic changes in sodium affect neural and muscle development in butterflies. Proceedings of the National Academy of Sciences, USA 111: 10221–10226. 10.1073/pnas.1323607111 PMC4104890 24927579

[nph71150-bib-0041] Wang X‐S , Yao Y , Liu X‐D . 2025. Sodium influences dietary breadth of an insect herbivore. Entomologia Generalis 45: 441–449.

[nph71150-bib-0042] War AR , Paulraj MG , Ahmad T , Buhroo AA , Hussain B , Ignacimuthu S , Sharma HC . 2012. Mechanisms of plant defense against insect herbivores. Plant Signaling & Behavior 7: 1306–1320. 22895106 10.4161/psb.21663 PMC3493419

[nph71150-bib-0043] Welti EAR , Kaspari M . 2021. Sodium addition increases leaf herbivory and fungal damage across four grasslands. Functional Ecology 35: 1212–1221.

[nph71150-bib-0044] Welti EAR , Sanders NJ , De Beurs KM , Kaspari M . 2019. A distributed experiment demonstrates widespread sodium limitation in grassland food webs. Ecology 100: e02600. 30726560 10.1002/ecy.2600

[nph71150-bib-0045] Wu H . 2018. Plant salt tolerance and Na^+^ sensing and transport. The Crop Journal 6: 215–225.

[nph71150-bib-0046] Xiao K , Shen K , Zhong J‐F , Li G‐Q . 2010. Effects of dietary sodium on performance, flight and compensation strategies in the cotton bollworm, *Helicoverpa armigera* (Hübner) (Lepidoptera: Noctuidae). Frontiers in Zoology 7: 11. 20385025 10.1186/1742-9994-7-11 PMC2859862

[nph71150-bib-0047] Yan L , Zhai Q , Wei J , Li S , Wang B , Huang T , Du M , Sun J , Kang L , Li C‐B *et al*. 2013. Role of tomato lipoxygenase D in wound‐induced jasmonate biosynthesis and plant immunity to insect herbivores. PLoS Genetics 9: e1003964. 24348260 10.1371/journal.pgen.1003964 PMC3861047

