# Supplementary material for: Tomato plants control leaf sodium levels to limit damage by Spodoptera littoralis larvae
Source: New Phytol. 2026 Apr 9;250(5):3417–27. doi: 10.1111/nph.71150 (PMC13150315; doi:10.1111/nph.71150)
Supplement: Supplementary file 1 — Fig. S1 Sodium concentration in the nutrient solution expressed in ppm during the growth cycle of Solanum lycopersicum plants in +Na and −Na treatments. Fig. S2 Total sodium content and its distribution in leaves, stem and root, in Solanum lycopersicum undamaged control +Na plants and in +Na plants damaged by Spodoptera littoralis larvae. Fig. S3 Plant physiological traits of Solanum lycopersicum plants grown under +Na and −Na treatments. Fig. S4 Plant biometric traits of Solanum lycopersicum plants grown under +Na and −Na treatments. Fig. S5 Expression analysis of SlPinI, SlPinII and SlProsys in Solanum lycopersicum leaves of undamaged +Na and −Na plants. Fig. S6 Expression analysis of SlProSys in Solanum lycopersicum leaves of +Na and −Na plants at 24, 48 and 72 h after Spodoptera littoralis infestation. Fig. S7 Expression analysis of SlPinI in Solanum lycopersicum leaves of +Na and −Na plants at 24, 48 and 72 h after Spodoptera littoralis infestation. Fig. S8 Expression analysis of SlPinII in Solanum lycopersicum leaves of +Na and −Na plants at 24, 48 and 72 h after Spodoptera littoralis infestation. Table S1 Primers used in this study. Table S2 Ion content of Solanum lycopersicum leaves grown under +Na and subjected to larvae attack. Table S3 Ion content of Solanum lycopersicum leaves grown under +Na vs −Na. Please note: Wiley is not responsible for the content or functionality of any Supporting Information supplied by the authors. Any queries (other than missing material) should be directed to the New Phytologist Central Office. [file NPH-250-3417-s001.docx]

**New Phytologist Supporting Information**

**Article title:** Tomato plants control leaf sodium levels to limit damage by *Spodoptera littoralis* larvae

**Authors:** Valerio Cirillo, Ilaria Di Lelio, Paola Punzo, Giovanni Jesu, Maria Giovanna De Luca, Marco Cepparulo, Claudio Russo, Nausicaa Pollaro, Antonio Marciano, Andrea Becchimanzi, Michael V. Mickelbart, Francesco Pennacchio, Albino Maggio

**Article acceptance date:** 9 March 2026

Figure S1 Sodium concentration in the nutrient solution expressed in ppm during the growth cycle of *Solanum lycopersicum* plants in +Na and −Na treatments.


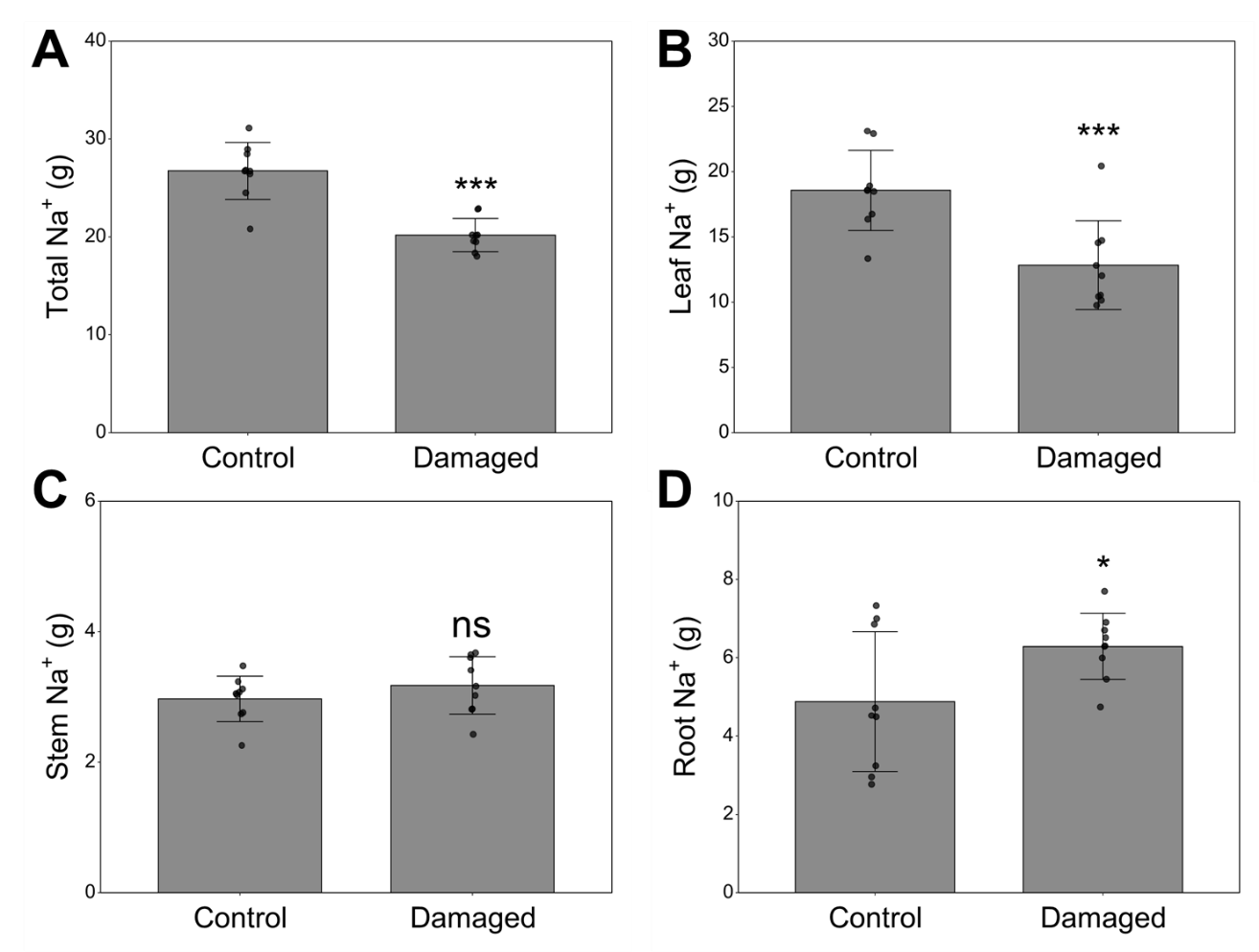


Figure S2 Total Sodium content (A) and its distribution in leaves (B), stem (C), and root (D) in *Solanum lycopersicum* undamaged +Na plants and in +Na plants damaged by *Spodoptera littoralis* larvae. Asterisks indicate significant differences between the two treatments according to Student’s t-test (*p < 0.05; ***p < 0.001; ns = not significant).


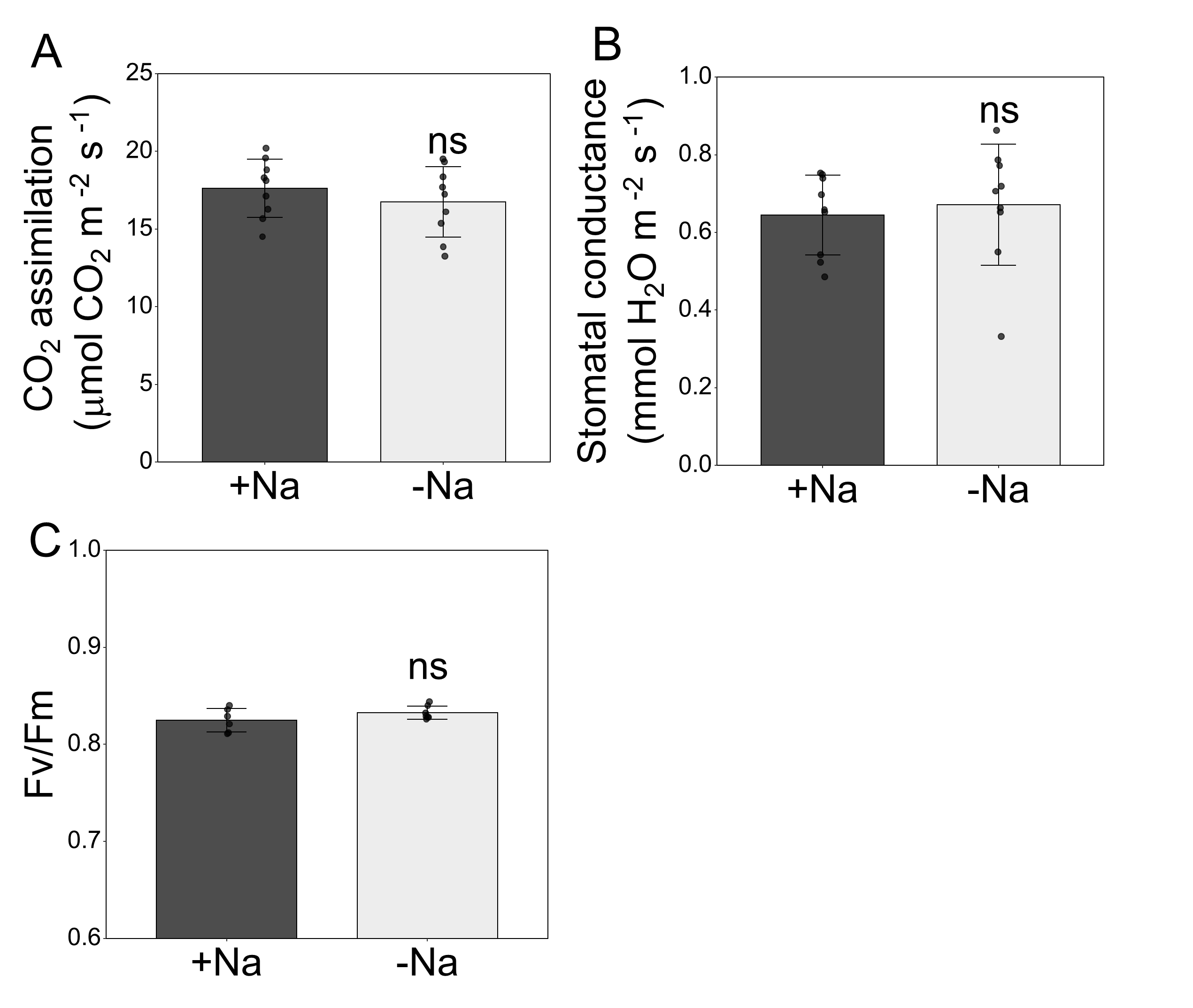


Figure S3 Plant physiological traits of *Solanum lycopersicum* plants grown under +Na and −Na treatments. Data reported are means ±SD of nine replicates. Statistical differences between the treatments have been tested by Student’s t-test (ns = not significant).


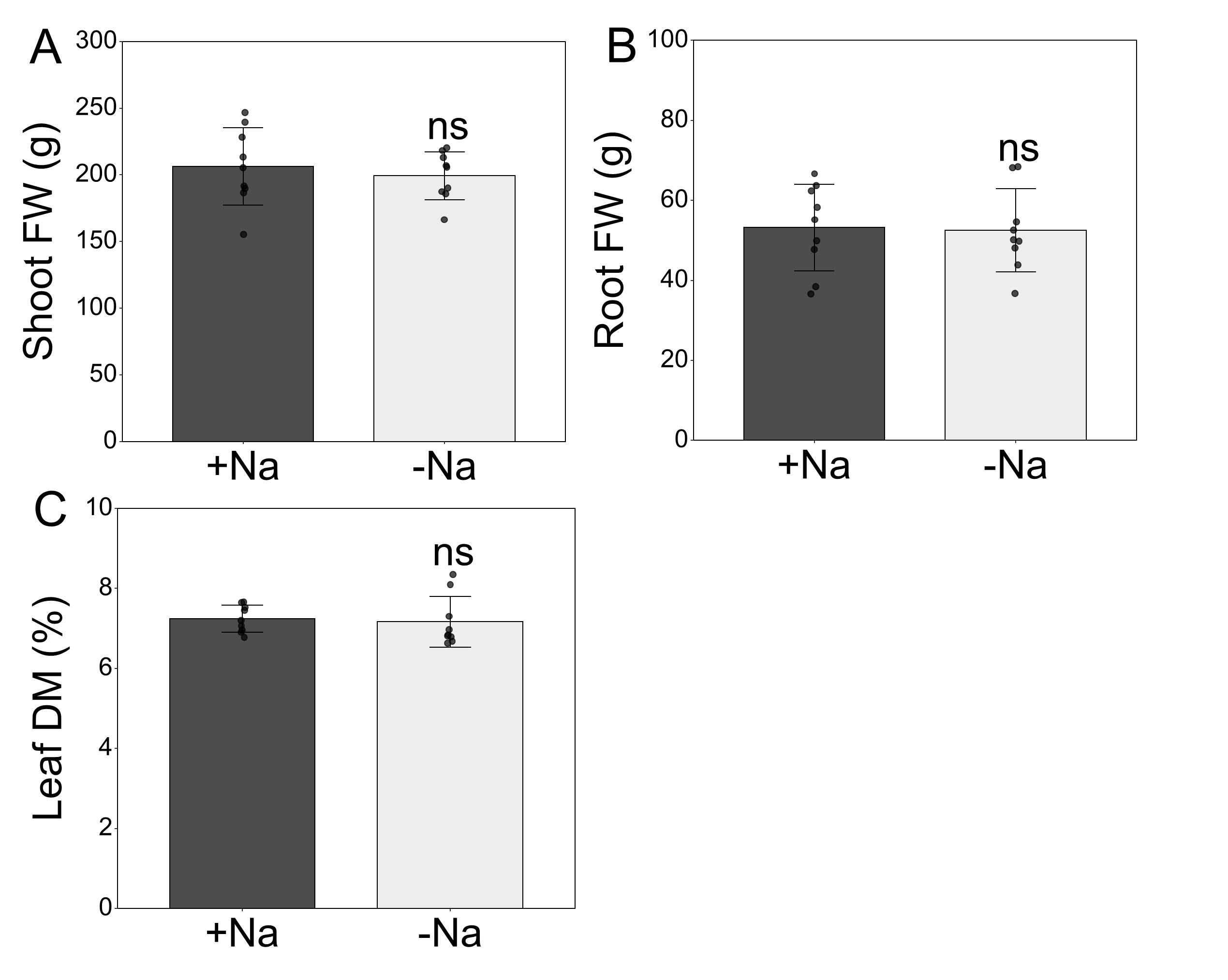


Figure S4 Plant biometric traits of *Solanum lycopersicum* plants grown under +Na and −Na treatments. Data reported are means ±SD of nine replicates. Statistical differences between the treatments have been tested by Student’s t-test (ns = not significant).


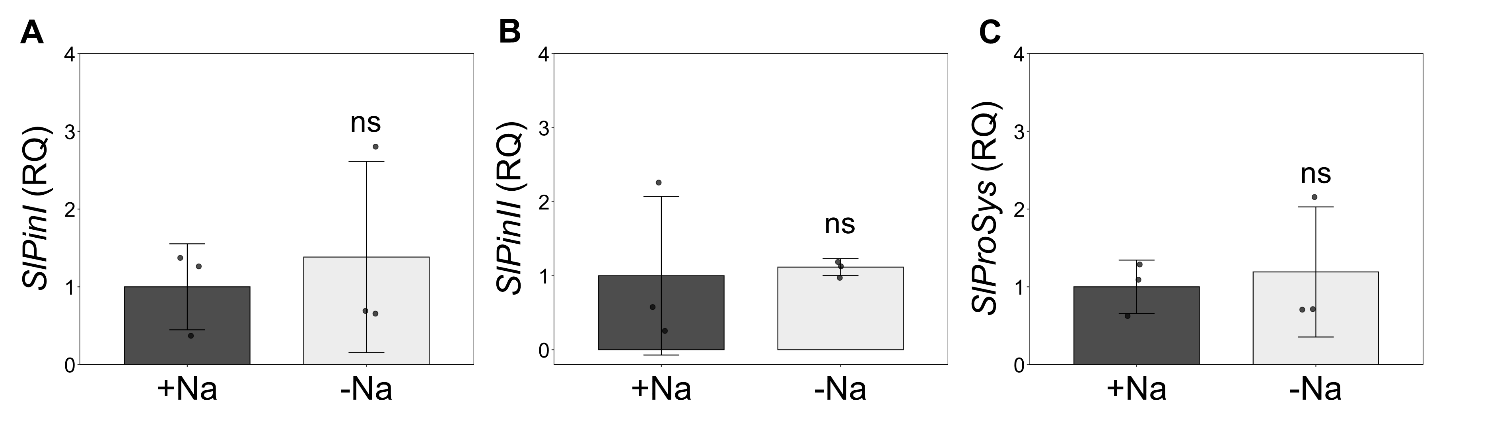


Figure S5 Expression analysis of *SlPinI* (A), *SlPinII* (B), and *SlProsys* (C) in *Solanum lycopersicum* leaves of undamaged +Na and −Na plants. Data were normalized using RNA from +Na plants. Data reported are means ±SD of three biological replicates. Asterisks indicate significant differences between the two treatments according to Student’s t-test (*p < 0.05; ***p < 0.001; ns = not significant).

*
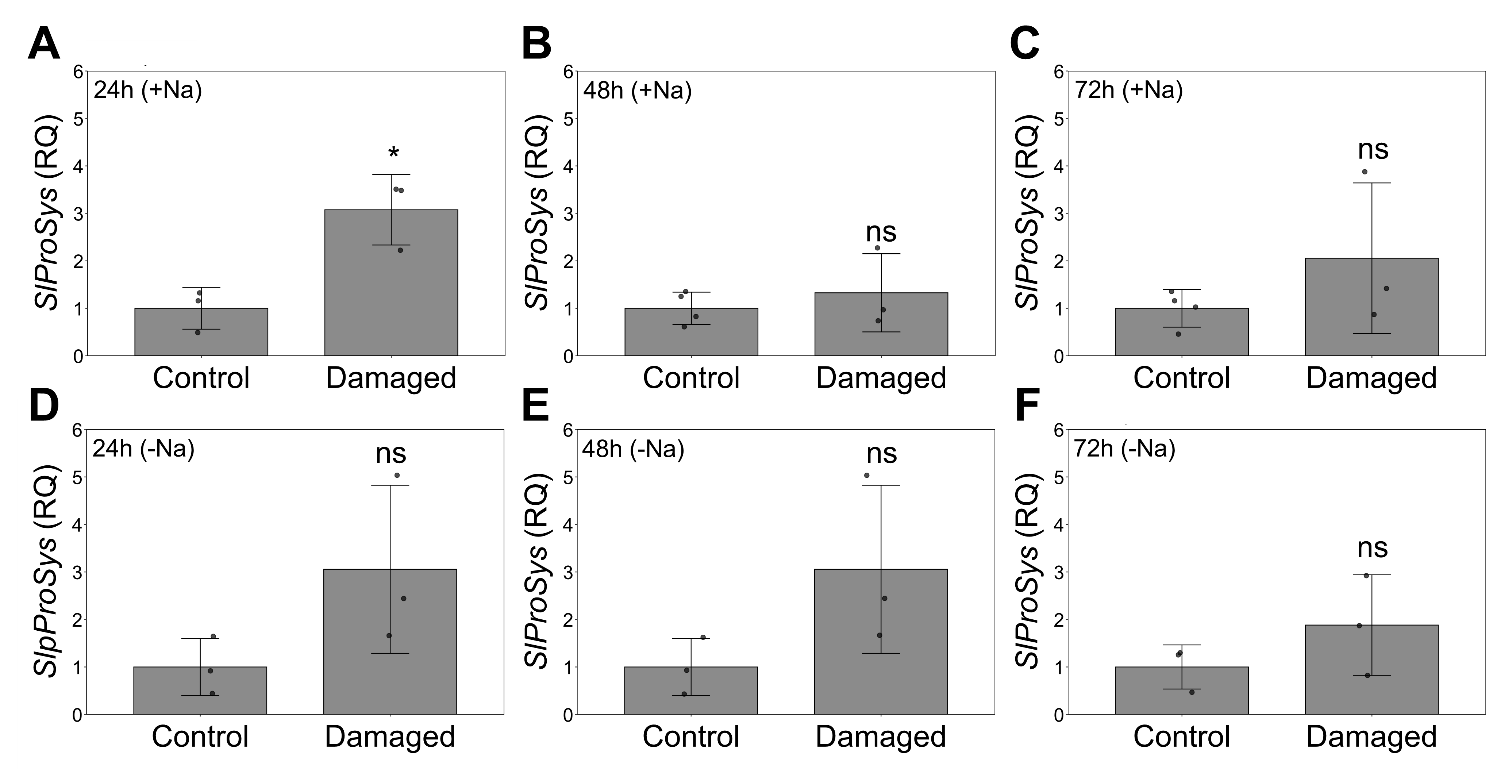
*

Figure S6 Expression analysis of *SlProSys* in *Solanum lycopersicum* leaves of +Na (A-B-C) and −Na plants (D-E-F) at 24, 48, and 72 h after *Spodoptera littoralis* infestation. Data were normalized using RNA from undamaged +Na plants. Data reported are means ±SD of three biological replicates. Asterisks indicate significant differences between the two treatments according to Student’s t-test (*p < 0.05; ***p < 0.001; ns = not significant).

*
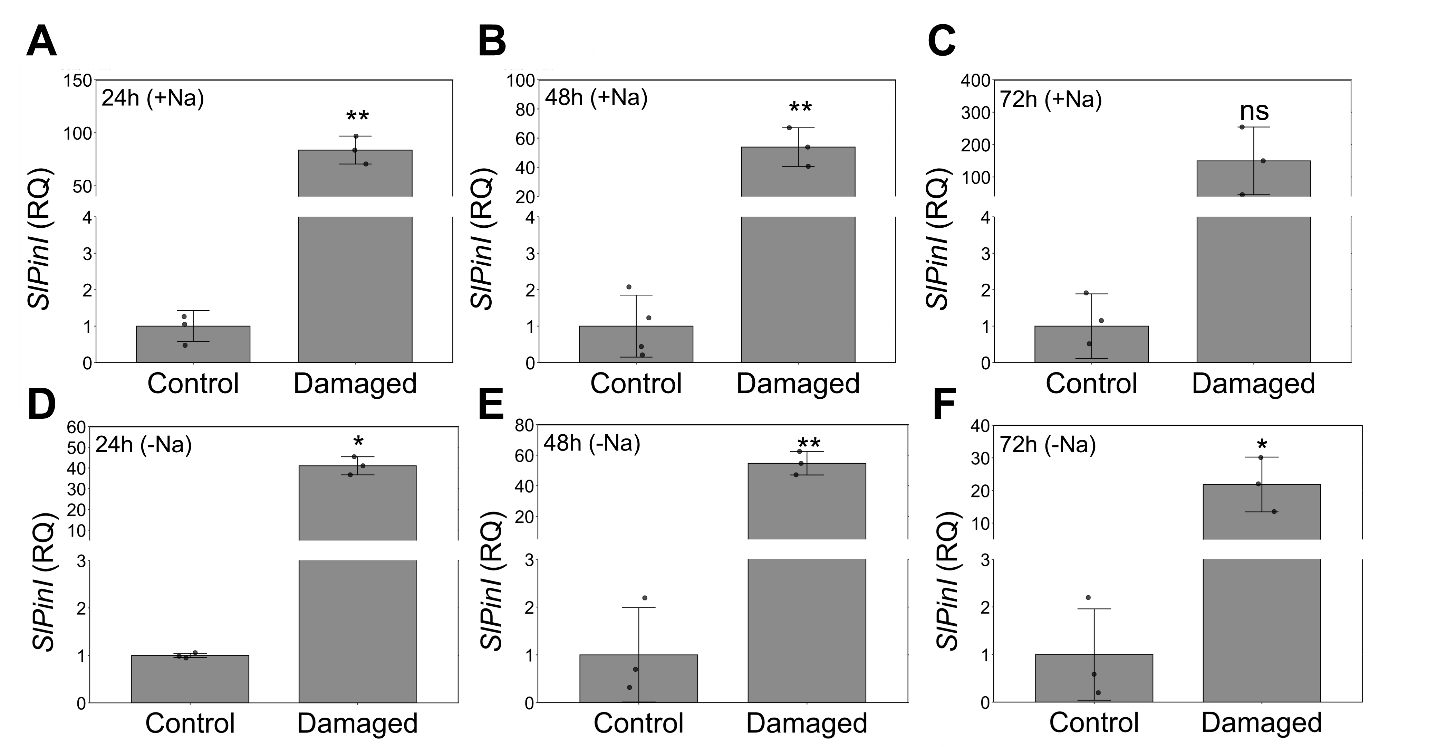
*

Figure S7 Expression analysis of *SlPinI* in *Solanum lycopersicum* leaves of +Na (A-B-C) and −Na plants (D-E-F) at 24, 48, and 72 h after *Spodoptera littoralis* infestation. Data were normalized using RNA from undamaged +Na plants. Data reported are means ±SD of three biological replicates. Asterisks indicate significant differences between the two treatments according to Student’s t-test (*p < 0.05; ***p < 0.001; ns = not significant).

*
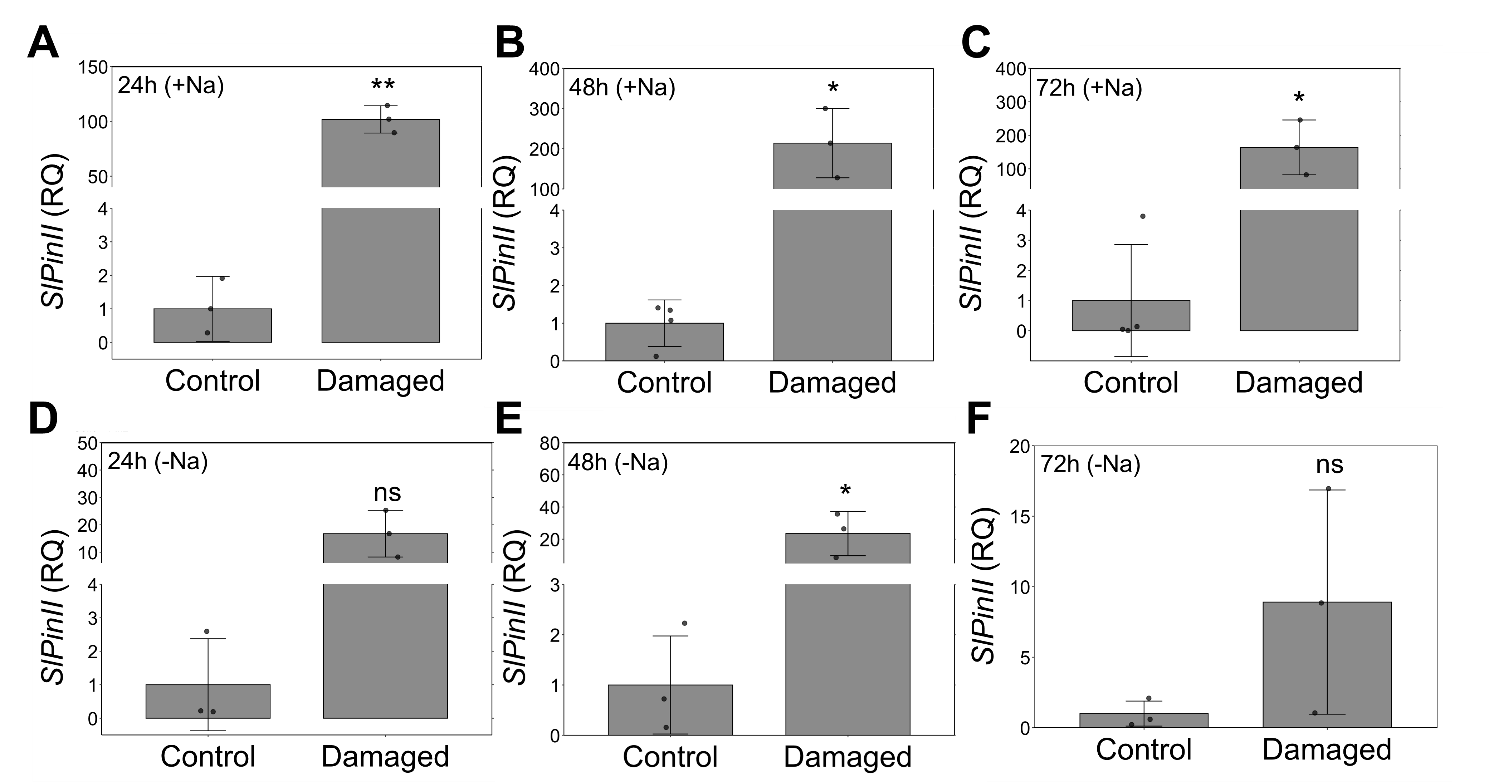
*

Figure S8 Expression analysis of *SlPinII* in *Solanum lycopersicum* leaves of +Na (A-B-C) and −Na plants (D-E-F) at 24, 48, and 72 h after *Spodoptera littoralis* infestation. Data were normalized using RNA from undamaged +Na plants. Data reported are means ±SD of three biological replicates. Asterisks indicate significant differences between the two treatments according to Student’s t-test (*p < 0.05; ***p < 0.001; ns = not significant).

Table S1 Primers used in this study

| **Name** | **Seq 5’-3’** |
| --- | --- |
| *SlEF1α* for | CTCCATTGGGTCGTTTTGCT |
| *SlEF1α* rev | GGTCACCTTGGCACCAGTTG |
| *SlHKT1;2* for | TGAGCTAGGGAATGTAATAAACG |
| *SlHKT1;2* rev | AGAGAGAAACTAACGATGAACC |
| *SlHKT1;1* for | TCTAGCCCAAGAAACTCAAAT |
| *SlHKT1;1* rev | CTAATGTTACAACTCCAAGGAATT |
| *SlSOS1* for | ACTGCCCGACAGGAACTAAA |
| *SlSOS1* rev | AACTTCCGTGCATCCTCTCC |
| *SlProSys* for | GGGAGGGTGCACTAGAAATA |
| *SlProSys* rev | TTGCATTTTGGGAGGATCAC |
| *SlPinI* for | GAAACTCTCATGGCACGAAAAG |
| *SlPinI* rev | CACCAATAAGTTCTGGCCACAT |
| *SlPinII* for | CCAAAAAGGCCAAATGCTTG |
| *SlPinII* rev | TGTGCAACACGTGGTACATCC |

Table S2 Ion content of *Solanum lycopersicum* leaves grown under +Na and subjected to larvae attack. Data reported are means ±SD of nine replicates. Statistical differences between the treatments have been tested by Student’s t-test (ns = not significant).

|  | **Potassium (ppm)** | **Calcium (ppm)** | **Nitrate (ppm)** |
| --- | --- | --- | --- |
| **Control** | 3528.6 | 822.9 | 4585.7 |
| **Damaged** | 3362.5 | 755.0 | 4350.0 |
| *p-value* | ns | ns | ns |

Table S3 Ion content of *Solanum lycopersicum* leaves grown under +Na vs −Na. Data reported are means ±SD of nine replicates. Statistical differences between the treatments have been tested by Student’s t-test (ns = not significant).

|  | **Sodium (ppm)** | **Potassium (ppm)** | **Calcium (ppm)** | **Nitrate (ppm)** |
| --- | --- | --- | --- | --- |
| **+Na** | 106.1 | 3528 | 822 | 4586 |
| **- Na** | 42.4 | 3220 | 895 | 4450 |
| *p-value* | *** | ns | ns | ns |
